# Early Diagnosis of Stevens-Johnson Syndrome in the Dental Clinic Setting

**DOI:** 10.7759/cureus.14160

**Published:** 2021-03-28

**Authors:** Wisam K Al Hathlol, Asma Almeslet

**Affiliations:** 1 Department of Pediatric Dentistry, National Guard Hospital, Dammam, SAU; 2 Oral Maxillofacial Surgery and Diagnosis Department, Riyadh Elm University, Riyadh, SAU

**Keywords:** stevens-johnson syndrome (sjs), dental emergency, skin rash, blisters, oral mucosal lesions, oral ulcers

## Abstract

Stevens-Johnson syndrome (SJS) is a life-threatening acute and fatal dermatological disease. It can present with many variations and rapidly worsens in a short period of time. Early diagnosis and management play an important role in stopping SJS from progression. Various drugs such as antibiotics, anticonvulsants and non-steroidal anti-inflammatory drugs can trigger the disease as an adverse effect. Oral and dental manifestation of SJS is uncommon. However, dentists should be clinically oriented towards signs and symptoms of the disease, both oral and systemic. We present a case of a 37-year-old male with no past medical history who presented to the dental emergency clinic complaining of dysphagia and burning sensation in the mouth. On oral examination, oral rash and blisters were observed. In addition, a bilateral forearm erythematous, non-blanching painful rash with some blisters was found after antibiotic administration three days earlier. The antibiotic was stopped and the patient was started on topical betamethasone for 14 days, topical chlorhexidine for 10 days, and oral nystatin suspension 100,000 units. A complete resolution of the oral and systemic manifestation was seen without the need for hospital admission.

## Introduction

Stevens-Johnson syndrome (SJS) is a life-threatening dermatological mucocutaneous illness that causes acute destruction of the epithelium of the skin due to an aggressive immune response [1,2]. Patients usually present with a spectrum of mucocutaneous exfoliative lesions involving the oral, ocular, or genital mucosa. Stevens-Johnson syndrome is part of a spectrum of diseases ranging from SJS when presented with less than 10% skin detachment to toxic epidermal necrolysis (TEN) when presented with greater than 30% skin detachment [3]. The more severe form of SJS is TEN and often overlaps with SJS. Clinical characteristic of SJS include hemorrhagic erosions, mucocutaneous tenderness and erosion of the mucous membrane, erythematous macules, blisters and denuded skin [2]. It commonly presents with skin rash involving oral mucosa or conjunctivae, but it could be life-threatening in rare cases [4]. It affects all individuals with genetic preposition such as age, gender and ethnicity. However, it has been reported that SJS occurs more often in old age and in women. Cases of SJS have been increasing as unfavorable drug reaction can occur by binding of the metabolite to the major histocompatibility complex (MHC) type 1 [4]. Furthermore, CD8+ cells, CD40 ligand cells and the innate immune system are also reported to play a role in the interaction with drugs causing SJS [4]. With the frequent use of prescribed medications such as antibiotics, anticonvulsants and nonsteroidal anti-inflammatory drugs (NSIADS), adverse drug reactions leading to SJS represent a continuing challenge for all healthcare providers.

## Case presentation

**Figure 1:**
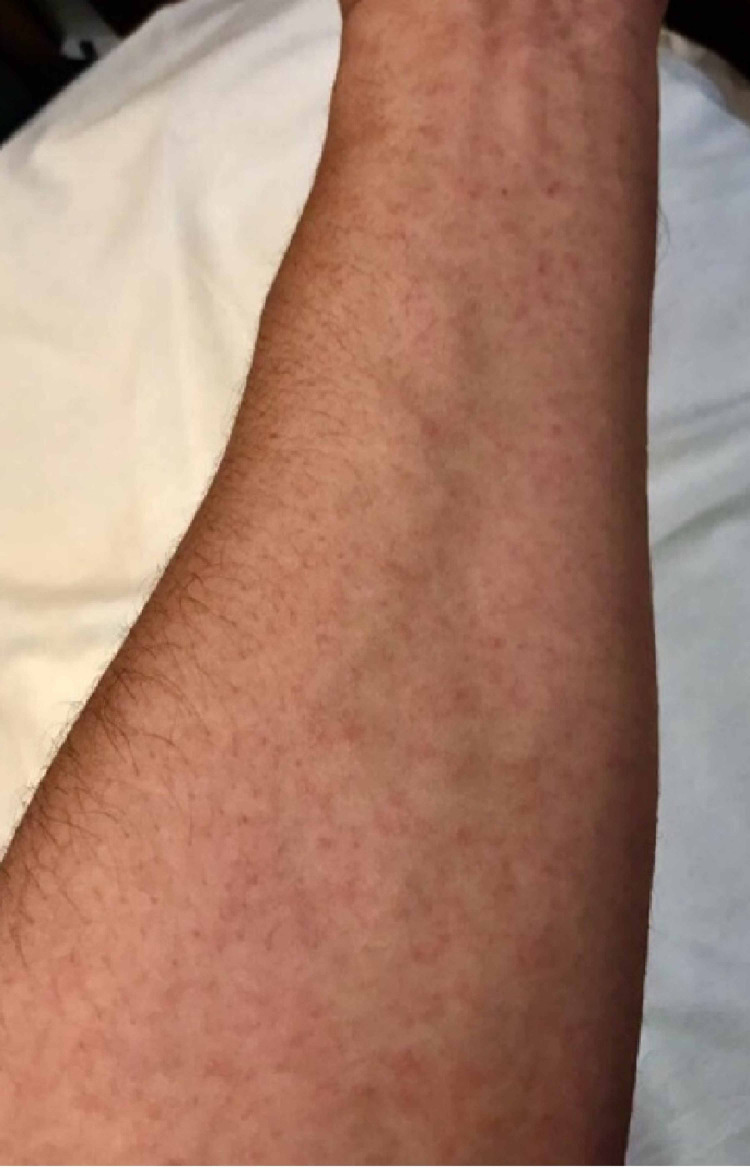
Skin rash over the patient's upper limb

A 37-year-old Saudi male presented to the dental emergency clinic of Riyadh Elm University complaining of burning sensation in the mouth and difficulty in swallowing and the condition was diagnosed as oral candidiasis. The symptoms were sudden and progressive starting five days earlier. At first, he went to a private clinic and was prescribed antibiotics after being diagnosed with a tooth abscess. The patient denied any use of other medications in the past. Afterwards, the symptoms did not subside and another episode of burning sensation and more difficulty in swallowing was accompanied with generalized erythematous non-blanching painful skin rash with very few blisters. Regarding his medical history, the patient was febrile for a duration of two days with overall joint pain and blurred vision with no known drug allergy. His family history was unremarkable for any similar conditions or allergy. On examination, he was febrile with a temperature of 40˚ C with enlarged cervical lymph nodes and skin rash over the upper limbs (Figure 1).

**Figure 2:**
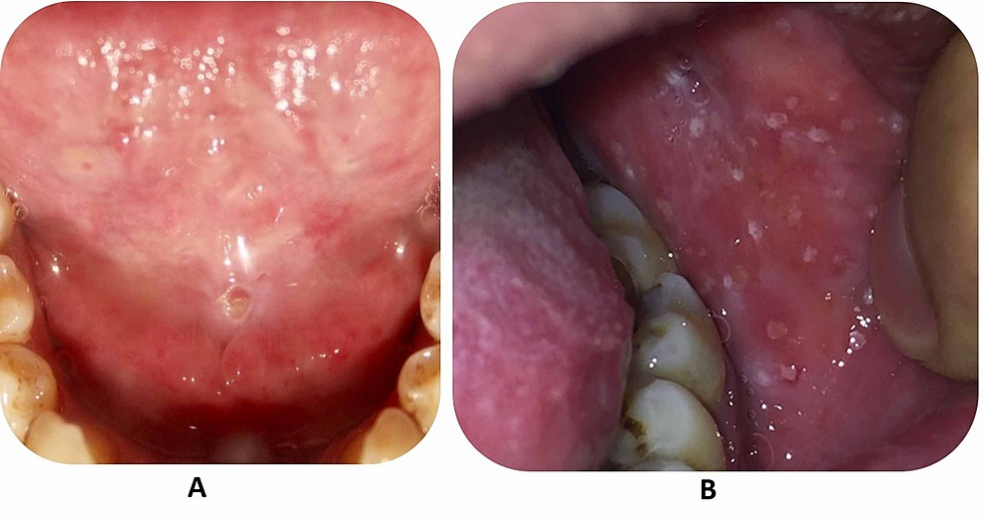
(A) Blisters on the floor of the mouth; (B) buccal mucosa with scattered oral ulcers with erythematous base

**Figure 3:**
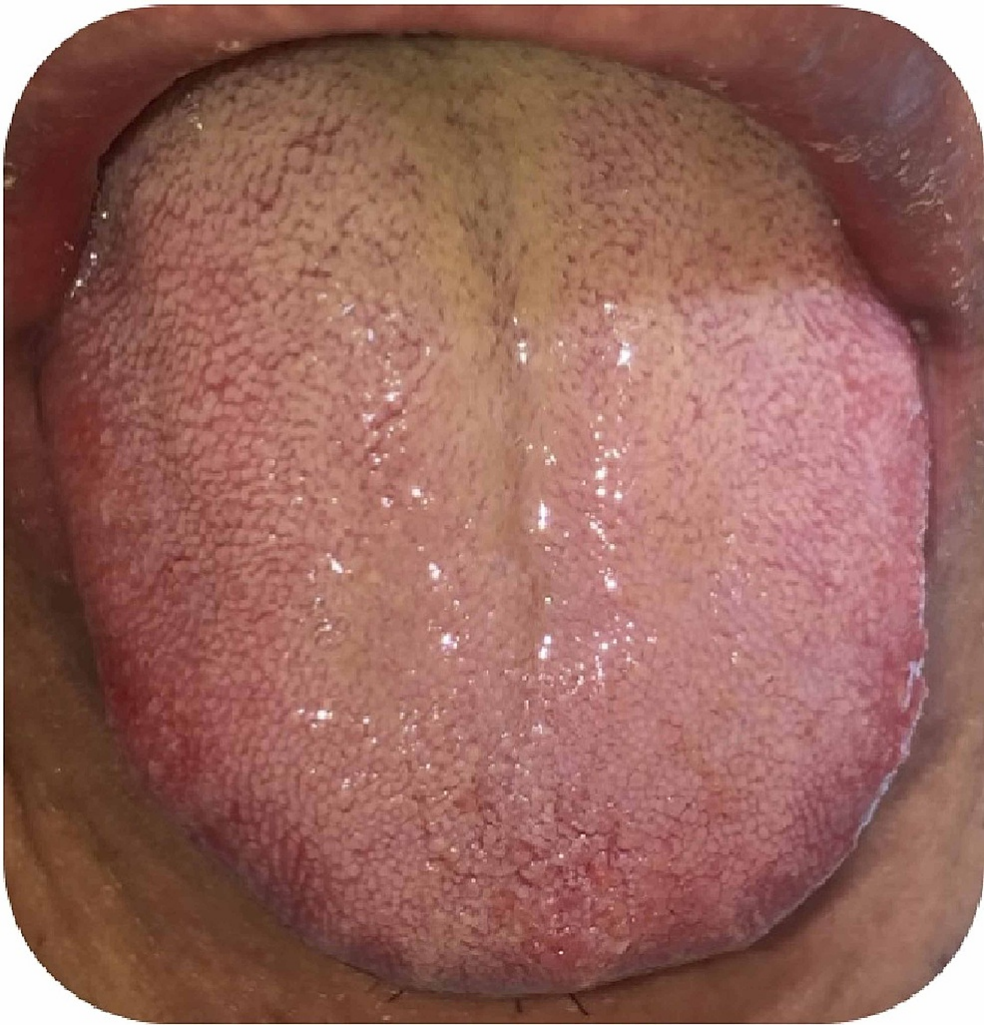
Coated tongue

On examination, there was no joint swelling, joint tenderness or itching. Local examination showed blisters on the floor of the mouth and buccal mucosa with scattered oral ulcers on an erythematous base (Figure 2), and coated tongue (Figure 3). Furthermore, on examination of the rash, lateral pressure on the skin resulted in shedding of the epidermis (positive Nikolsky sign).

**Figure 4:**
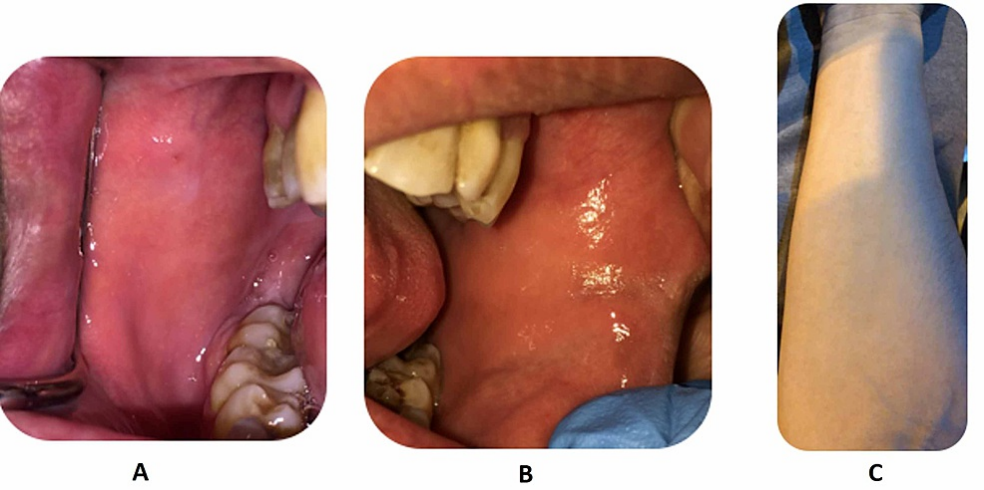
Buccal mucosa of the (A) right and (B) left cheek; (C) forearm showing no signs of rash; three weeks after treatment, the patient showed improved mucocutaneous lesions

Suspicion of herpes simplex virus (HSV), bullous pemphigoid, human immunodeficiency virus (HIV) or autoimmune vasculitis was raised. He was started treatment with topical betamethasone for 14 days, topical chlorhexidine for 10 days and oral nystatin suspension 100,000 units. Laboratory investigations showed negative results in pan cultures, and HIV and hepatitis screening. The patient was discharged and was reviewed three weeks later in our clinic with no signs of fever or signs of active disease in the mouth, and improvement in general and local clinical manifestations (Figure 4). He was referred to an infectious disease department for a second opinion in a different hospital. Due to the clinical picture, diagnosis of mild Steven-Johnson syndrome was established while immuno-deficiency disorders were excluded as it did not fit with the presentation of patient's history.

## Discussion

This case report highlights the early clinical features of SJS and also stresses the importance of its early recognition even in the dental setting. SJS results from an extensive keratinocyte apoptosis, or programmed cell death, which is provoked by a cell-mediated cytotoxic reaction involving T lymphocytes and natural killer cells [5]. Studies have shown that the concentration of granulysin in patient blisters correlates with the severity of the disease [6]. Furthermore, granulysin that is produced by blisters has been shown to be sufficient to induce keratinocyte cell death and blistering in the skin in a dose-dependent manner [7]. Another theory is that the drug reacts with the major histocompatibility complex that is responsible for the activation of cytotoxic T lymphocytes. Consequently, this process induces apoptosis in keratinocytes throughout the entire depth of the dermis resulting in painful blistering of the skin and mucous membranes [8].

The management of SJS is challenging requiring several clinicians who would usually be involved in the care of these patients including a dermatologist, an intensivist, a pulmonologist, nephrologist, an ophthalmologist, a plastic surgeon and a gastroenterologist. The essential treatment of these patients is cessation of the causative drug with fluid replacement, hospital admission and pain killers [4]. However, we could not admit the patient as it was a dental clinic and he was transferred accordingly to a different hospital. Even though our patient did not present with severe oral hemorrhagic manifestation, his rapid progression might have led to serious complications. Therefore, early diagnosis and management of such cases are often crucial in preventing rapid deterioration that may lead to intubation and intensive care unit admission [9]. In addition, the pharmacist should closely monitor the medications that the patient is using to prevent exacerbation of the condition [10,11].

The management outcome of patients with SJS depends on the extent and severity of skin involvement. For example, in those patients with a mild eruption, the lesions usually improve in 12 to 16 weeks. Mild scarring may appear, but there is usually no systemic functional loss unless the eyes and other mucous membranes are involved. It has been reported that the mortality rate in SJS is 1%-27% and increases up to 20% if there is skin involvement [4]. Furthermore, the mortality rate is worse if it is accompanied by bacterial infection. Additional risk factors that adversely affect the outcome include advanced age, leucopenia, presence of a malignancy, renal dysfunction and hyperglycemia [12,13].

SJS is still an ongoing challenge for emergency department care providers, and misdiagnosis of this condition may lead to severe consequences. Thus, recently it has been suggested that clinicians need to be familiar with the clinical features of SJS to facilitate prompt recognition and appropriate care [14]. Obviously, this rare condition would be much more challenging for dental practitioners due to the rarity of presentations and dental orientation. Obtaining a thorough medical history and performing a proper examination including oral health assessment is important in SJS. In addition, skin exposure is essential for early diagnosis and management of this condition.

Our case is unique due to the early recognition of this severe syndrome by a dental clinician and transferring the patient to an appropriate level of care without the need of hospitalization. Thus, this case is emphasizing the need for dental practitioners to be aware of early recognition of SJS, and to enable them to promptly manage this critical clinical condition, and thus avoid any adverse outcome.

## Conclusions

Stevens-Johnson syndrome is a rare, acute and fatal mucocutaneous dermatological disease. It presents commonly with several risk factors including medications such as antibiotics. Dentists should be aware of this rare illness and should perform a comprehensive evaluation for any patient attending the dental clinic with oral lesions to facilitate early recognition and appropriate care.
